# (*E*)-3,5-Dimethyl-1-*p*-tolyl-4-(*p*-tolyl­diazen­yl)-1*H*-pyrazole

**DOI:** 10.1107/S1600536812000360

**Published:** 2012-01-11

**Authors:** Carlos Bustos, Marcia Pérez-Cerda, Luis Alvarez-Thon, Enrique Barrales-Salcedo, Maria Teresa Garland

**Affiliations:** a Instituto de Ciencias Químicas, Universidad Austral de Chile, Avda. Los Robles s/n, Campus Isla Teja, Casilla 567, Valdivia, Chile; b Departamento de Ciencias Físicas, Universidad Andres Bello, Avda. República 220, Santiago de Chile, Chile; c Laboratorio de Cristalografía, Departamento de Física, Facultad de Ciencias Físicas y Matemáticas, Universidad de Chile, Av. Blanco Encalada 2008, Santiago de Chile, Chile

## Abstract

There are two independent mol­ecules, *A* and *B*, in the asymmetric unit of the title compound, C_19_H_20_N_4_, in each of which the N=N double bond has an *E* conformation. The dihedral angles between the pyrazole ring and the *p*-tolyl rings in the 1- and 4-positions are 22.54 (8) and 35.73 (7)°, respectively, in mol­ecule *A*. The corresponding dihedral angles in mol­ecule *B* are 28.13 (8) and 22.18 (8)°. In the crystal, the *A* and *B* mol­ecules are linked by weak C—H⋯π inter­actions, leading to inversion dimers in each case.

## Related literature

For related syntheses, see: Bustos *et al.* (2007 ▶, 2009 ▶). For the biological activity of compounds with pyrazole nuclei, see: Card *et al.* (2005 ▶); Daidone *et al.* (1998 ▶); Devi *et al.* (1983 ▶); Eid *et al.* (1978 ▶); El-Emary & Bakhite (1999 ▶); Elguero *et al.* (2002 ▶); Habit & Tawil (1981 ▶); Haufel & Breitmaier (1974 ▶); Menozzi *et al.* (1997 ▶); Pathak & Bahel (1980 ▶); Penning *et al.* (1997 ▶); Rathelot *et al.* (1995 ▶); Tedlaouti *et al.* (1990 ▶, 1991 ▶); Terrett *et al.* (1996 ▶); Wustrow *et al.* (1998 ▶). For related structures, see: Duprez & Heumann (2004 ▶); Rojas *et al.* (2004 ▶).
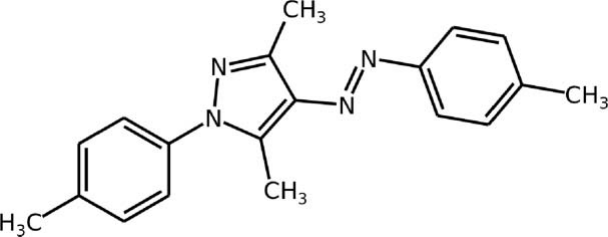


## Experimental

### 

#### Crystal data

C_19_H_20_N_4_
*M* *_r_* = 304.39
Monoclinic, 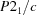

*a* = 9.4320 (8) Å
*b* = 19.1552 (17) Å
*c* = 18.4511 (16) Å
β = 101.931 (1)°
*V* = 3261.6 (5) Å^3^
*Z* = 8
Mo *K*α radiation
μ = 0.08 mm^−1^
*T* = 150 K
0.37 × 0.20 × 0.15 mm

#### Data collection

Bruker D8 Discover with a SMART CCD area-detector diffractometer
25935 measured reflections
6641 independent reflections
4272 reflections with *I* > 2σ(*I*)
*R*_int_ = 0.044

#### Refinement

*R*[*F* ^2^ > 2σ(*F* ^2^)] = 0.046
*wR*(*F* ^2^) = 0.121
*S* = 0.90
6641 reflections
423 parameters
H-atom parameters constrained
Δρ_max_ = 0.24 e Å^−3^
Δρ_min_ = −0.26 e Å^−3^

### 

Data collection: *SMART* (Bruker, 2001 ▶); cell refinement: *SAINT* (Bruker, 2000 ▶); data reduction: *SAINT*; program(s) used to solve structure: *SHELXS97* (Sheldrick, 2008 ▶); program(s) used to refine structure: *SHELXL97* (Sheldrick, 2008 ▶); molecular graphics: *XP* in *SHELXTL-PC* (Sheldrick, 2008 ▶); software used to prepare material for publication: *PLATON* (Spek, 2009 ▶) and *Mercury* (Macrae *et al.*, 2006 ▶).

## Supplementary Material

Crystal structure: contains datablock(s) global, I. DOI: 10.1107/S1600536812000360/lx2212sup1.cif

Structure factors: contains datablock(s) I. DOI: 10.1107/S1600536812000360/lx2212Isup2.hkl

Supplementary material file. DOI: 10.1107/S1600536812000360/lx2212Isup3.cml

Additional supplementary materials:  crystallographic information; 3D view; checkCIF report

## Figures and Tables

**Table 1:** Hydrogen-bond geometry (Å, °) *Cg*1 and *Cg*2 are the centroids of the C33–C38 benzene ring and the N4/N3/C9–C11 pyrazole ring, respectively.

*D*—H⋯*A*	*D*—H	H⋯*A*	*D*⋯*A*	*D*—H⋯*A*
C7—H7⋯*Cg*1^i^	0.95	2.76	3.4847 (18)	133
C27—H27*A*⋯*Cg*2^ii^	0.98	2.72	3.6322 (18)	156

Symmetry codes: (i) 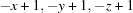; (ii) 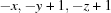.

**Fig. 1.**
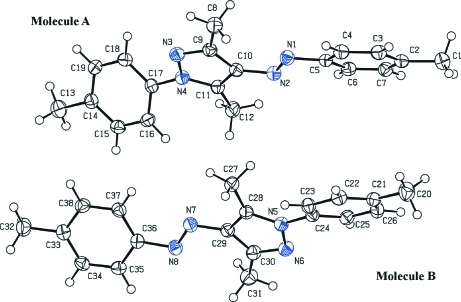
The molecular structure of the title compound with the atom numbering scheme. Displacement ellipsoids are drawn at the 50% probability level. H atoms are presented as small spheres of arbitrary radius.

**Fig. 2.**
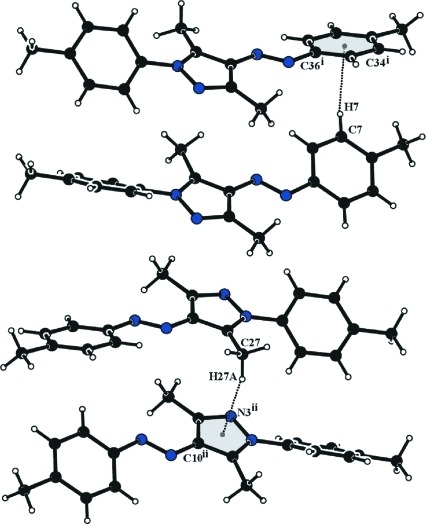
A view of the C–H···π interactions (dotted lines) in the crystal structure of the title compound. [Symmetry codes: (i) - x +1, - y + 1, - z + 1; (ii) - x, - y + 1, - z + 1].

## supplementary crystallographic information

### Comment

Pyrazole nuclei are important targets in the pharmaceutical industry (Elguero *et al.*, 2002) because they are the core of numerous biologically active compounds including blockbuster drugs such as Celebrex (Penning *et al.*, 1997) and Viagra (Terrett *et al.*, 1996). Besides, they have also pharmacological interest as anti-anxiety (Haufel & Breitmaier, 1974; Wustrow *et al.*, 1998), antipyretic, analgesic and anti-inflammatory drugs (Eid *et al.*, 1978; Menozzi *et al.*, 1997; Penning *et al.*, 1997). On the other hand, they show antimicrobial (Habit & Tawil, 1981; Pathak & Bahel, 1980; Devi *et al.*, 1983; Daidone *et al.*, 1998; El-Emary & Bakhite, 1999) and anti-parasitic activities in the N-heterocyclic series (Rathelot *et al.*, 1995; Tedlaouti *et al.*, 1990; Tedlaouti *et al*., 1991). Compounds from a family of pyrazole related derivatives have been described as potent PDE4B or PDE4D inhibitors (Card *et al.*, 2005). Pyrazole compounds have been used for some time as ligands in transition metal chemistry, since the heterocyclic nucleus may coordinate the metal directly *via* one or both vicinal N atoms and some of these compounds present catalytic activity (Rojas *et al.*, 2004). Moreover, it is known that the metal may be bound to several pyrazole nuclei, to yield polypyrazole systems linked to the heteroatoms and/or C atoms (Duprez & Heumann, 2004). In this work we present the crystal structure of the title compound, which was prepared following a previously reported similar procedure (Bustos *et al*., 2007; Bustos *et al.*, 2009).

The title compound shown in Fig. 1, crystallizes in the monoclinic *P*2_1_/*c* space group with two independent molecules in the asymmetric unit, displaying an *E* configuration with closely comparable conformations (r.m.s. overlay = 0.18 Å for non-H atoms). The dihedral angles between the pyrazole ring and the 1-(*p*-tolyl) and 4-(*p*-tolyl) rings are 22.54 (8) and 35.73 (7)°, in molecule A, respectively. The dihedral angles formed by the pyrazole ring and the 1-(*p*-tolyl) and 4-(*p*-tolyl) rings are 28.50 (8)° and 21.79 (8)°, in molecule B, respectively. In the crystal packing (Fig. 2), the A and B molecules are linked by weak intermolecular C–H···π interactions; the first one between an H atom of the 4-(*p*-tolyl) ring and the 4-(*p*-tolyl) ring (Table 1, first entry; Cg1 is the centroid of C33–C38 benzene ring), and the second one between a methyl H atom attached to the pyrazole ring and the pyrazole ring (Table 1, second entry; Cg2 is the centroid of the N4/N3/C9-C11 pyrazole ring).

### Experimental

In a 100 ml round-bottomed flask were added 2.16 g (9.9 mmole) of 3-(2-*p*-tolylhydrazinylidene)pentane-2,4-dione, 1.65 g (10.4 mmole) *p*-tolylhydrazine hydrochloride, 5 ml of glacial acetic acid and 30 ml of ethanol. The reaction mixture was magnetically stirred and heated under reflux during 36 hrs. Then, after cooling at room temperature, the yellow precipitate was filtrated by suction and dried in a vacuum oven at 40°C during 24 hrs. Yield 78% of crude product. Single crystals suitable for X-ray studies were obtained by crystallization from a 1:1 ethanol/acetone mixture. Melting point: 137-138 °C.

### Refinement

All H atoms were placed in geometrically idealized positions and constrained to ride on their parent atoms, with aromatic C–H = 0.95 Å, methyl C–H = 0.98 Å and *U*_iso_(H) = 1.2*U*_eq_(aromatic C) or *U*_iso_(H) = 1.5*U*_eq_(methyl C).

### Crystal data

**Table:**

C_19_H_20_N_4_	*F*(000) = 1296
*M**_r_* = 304.39	*D*_x_ = 1.240 Mg m^−^^3^
Monoclinic, *P*2_1_/*c*	Mo *K*α radiation, λ = 0.71073 Å
Hall symbol: -P 2ybc	Cell parameters from 999 reflections
*a* = 9.4320 (8) Å	θ = 2.1–26.3°
*b* = 19.1552 (17) Å	µ = 0.08 mm^−^^1^
*c* = 18.4511 (16) Å	*T* = 150 K
β = 101.931 (1)°	Block, yellow
*V* = 3261.6 (5) Å^3^	0.37 × 0.20 × 0.15 mm
*Z* = 8	

### Data collection

**Table:**

Bruker D8 Discover with a SMART CCD area-detector diffractometer	4272 reflections with *I* > 2σ(*I*)
Radiation source: fine-focus sealed tube	*R*_int_ = 0.044
graphite	θ_max_ = 26.3°, θ_min_ = 2.1°
Detector resolution: 10.0 pixels mm^-1^	*h* = −11→11
φ and ω scans	*k* = −23→23
25935 measured reflections	*l* = −23→23
6641 independent reflections	

### Refinement

**Table:**

Refinement on *F*^2^	Primary atom site location: structure-invariant direct methods
Least-squares matrix: full	Secondary atom site location: difference Fourier map
*R*[*F*^2^ > 2σ(*F*^2^)] = 0.046	Hydrogen site location: difference Fourier map
*wR*(*F*^2^) = 0.121	H-atom parameters constrained
*S* = 0.90	*w* = 1/[σ^2^(*F*_o_^2^) + (0.0649*P*)^2^] where *P* = (*F*_o_^2^ + 2*F*_c_^2^)/3
6641 reflections	(Δ/σ)_max_ < 0.001
423 parameters	Δρ_max_ = 0.24 e Å^−^^3^
0 restraints	Δρ_min_ = −0.26 e Å^−^^3^

### Special details

**Table:**

Geometry. All esds (except the esd in the dihedral angle between two l.s. planes) are estimated using the full covariance matrix. The cell esds are taken into account individually in the estimation of esds in distances, angles and torsion angles; correlations between esds in cell parameters are only used when they are defined by crystal symmetry. An approximate (isotropic) treatment of cell esds is used for estimating esds involving l.s. planes.
Refinement. Refinement of F^2^ against ALL reflections. The weighted R-factor wR and goodness of fit S are based on F^2^, conventional R-factors R are based on F, with F set to zero for negative F^2^. The threshold expression of F^2^ > 2sigma(F^2^) is used only for calculating R-factors(gt) etc. and is not relevant to the choice of reflections for refinement. R-factors based on F^2^ are statistically about twice as large as those based on F, and R- factors based on ALL data will be even larger.

### Fractional atomic coordinates and isotropic or equivalent isotropic displacement parameters (Å^2^)

**Table:**

	*x*	*y*	*z*	*U*_iso_*/*U*_eq_	
C1	0.62673 (19)	0.86483 (8)	0.38328 (9)	0.0404 (4)	
H1A	0.7211	0.8655	0.4176	0.061*	
H1B	0.5578	0.8934	0.4034	0.061*	
H1C	0.6369	0.8837	0.3353	0.061*	
C2	0.57203 (17)	0.79090 (8)	0.37339 (8)	0.0294 (4)	
C3	0.42693 (17)	0.77661 (8)	0.34211 (9)	0.0309 (4)	
H3	0.3614	0.8141	0.3276	0.037*	
C4	0.37747 (17)	0.70868 (8)	0.33201 (9)	0.0302 (4)	
H4	0.2787	0.6998	0.3103	0.036*	
C5	0.47198 (16)	0.65319 (8)	0.35359 (8)	0.0257 (4)	
C6	0.61738 (16)	0.66681 (8)	0.38429 (8)	0.0289 (4)	
H6	0.6832	0.6293	0.3983	0.035*	
C7	0.66515 (17)	0.73470 (8)	0.39426 (8)	0.0297 (4)	
H7	0.7640	0.7434	0.4159	0.036*	
C8	0.20938 (17)	0.48098 (8)	0.24859 (9)	0.0358 (4)	
H8A	0.2579	0.5048	0.2134	0.054*	
H8B	0.1594	0.5155	0.2736	0.054*	
H8C	0.1387	0.4476	0.2219	0.054*	
C9	0.31931 (16)	0.44308 (8)	0.30478 (8)	0.0277 (4)	
C10	0.43929 (16)	0.46954 (8)	0.35718 (8)	0.0275 (4)	
C11	0.50928 (17)	0.41225 (8)	0.39333 (9)	0.0280 (4)	
C12	0.64728 (17)	0.40997 (8)	0.45042 (9)	0.0349 (4)	
H12A	0.6249	0.4062	0.4998	0.052*	
H12B	0.7027	0.4528	0.4475	0.052*	
H12C	0.7046	0.3695	0.4413	0.052*	
C13	0.5456 (2)	0.06412 (8)	0.41684 (10)	0.0429 (5)	
H13A	0.6297	0.0582	0.4576	0.064*	
H13B	0.5658	0.0426	0.3718	0.064*	
H13C	0.4609	0.0416	0.4298	0.064*	
C14	0.51564 (17)	0.14079 (8)	0.40347 (9)	0.0301 (4)	
C15	0.54384 (16)	0.18874 (8)	0.46061 (9)	0.0294 (4)	
H15	0.5840	0.1727	0.5093	0.035*	
C16	0.51557 (16)	0.25916 (8)	0.44941 (8)	0.0285 (4)	
H16	0.5337	0.2907	0.4900	0.034*	
C17	0.46036 (15)	0.28334 (8)	0.37817 (8)	0.0255 (4)	
C18	0.42990 (17)	0.23632 (8)	0.31976 (9)	0.0306 (4)	
H18	0.3910	0.2525	0.2710	0.037*	
C19	0.45630 (17)	0.16604 (8)	0.33258 (9)	0.0335 (4)	
H19	0.4337	0.1342	0.2924	0.040*	
C20	−0.0388 (2)	0.93105 (8)	0.58778 (10)	0.0443 (5)	
H20A	−0.1275	0.9382	0.5502	0.066*	
H20B	0.0436	0.9517	0.5707	0.066*	
H20C	−0.0492	0.9533	0.6342	0.066*	
C21	−0.01315 (18)	0.85406 (8)	0.60040 (9)	0.0310 (4)	
C22	−0.12431 (17)	0.80610 (8)	0.58325 (9)	0.0313 (4)	
H22	−0.2188	0.8221	0.5613	0.038*	
C23	−0.10280 (16)	0.73558 (8)	0.59691 (9)	0.0293 (4)	
H23	−0.1822	0.7041	0.5860	0.035*	
C24	0.03503 (16)	0.71101 (8)	0.62659 (8)	0.0252 (3)	
C25	0.14958 (16)	0.75778 (8)	0.64444 (8)	0.0293 (4)	
H25	0.2444	0.7414	0.6650	0.035*	
C26	0.12464 (17)	0.82825 (8)	0.63203 (9)	0.0330 (4)	
H26	0.2030	0.8600	0.6453	0.040*	
C27	−0.13084 (17)	0.58129 (8)	0.54119 (9)	0.0320 (4)	
H27A	−0.2230	0.5878	0.5570	0.048*	
H27B	−0.1322	0.5366	0.5152	0.048*	
H27C	−0.1170	0.6194	0.5079	0.048*	
C28	−0.00930 (16)	0.58146 (8)	0.60756 (8)	0.0267 (4)	
C29	0.05751 (16)	0.52430 (8)	0.64582 (8)	0.0271 (4)	
C30	0.17037 (16)	0.55115 (8)	0.70209 (9)	0.0296 (4)	
C31	0.28088 (17)	0.51354 (9)	0.75777 (9)	0.0384 (4)	
H31A	0.3512	0.5471	0.7844	0.058*	
H31B	0.3310	0.4793	0.7325	0.058*	
H31C	0.2331	0.4894	0.7930	0.058*	
C32	−0.09013 (19)	0.12593 (8)	0.59920 (10)	0.0405 (4)	
H32A	−0.0023	0.1010	0.5936	0.061*	
H32B	−0.1624	0.1233	0.5527	0.061*	
H32C	−0.1293	0.1045	0.6391	0.061*	
C33	−0.05381 (16)	0.20119 (8)	0.61788 (9)	0.0295 (4)	
C34	0.02167 (16)	0.22054 (8)	0.68803 (9)	0.0300 (4)	
H34	0.0494	0.1858	0.7250	0.036*	
C35	0.05680 (16)	0.28949 (8)	0.70455 (8)	0.0293 (4)	
H35	0.1087	0.3017	0.7526	0.035*	
C36	0.01679 (16)	0.34114 (8)	0.65136 (8)	0.0260 (4)	
C37	−0.05932 (16)	0.32253 (8)	0.58129 (9)	0.0286 (4)	
H37	−0.0880	0.3575	0.5446	0.034*	
C38	−0.09334 (16)	0.25366 (8)	0.56474 (9)	0.0302 (4)	
H38	−0.1445	0.2415	0.5165	0.036*	
N1	0.41335 (14)	0.58491 (6)	0.33852 (7)	0.0294 (3)	
N2	0.49150 (14)	0.53715 (7)	0.37309 (7)	0.0294 (3)	
N3	0.31487 (13)	0.37412 (7)	0.30873 (7)	0.0302 (3)	
N4	0.43238 (13)	0.35578 (7)	0.36404 (7)	0.0271 (3)	
N5	0.06006 (13)	0.63840 (7)	0.64137 (7)	0.0273 (3)	
N6	0.17222 (14)	0.62012 (7)	0.69907 (7)	0.0310 (3)	
N7	0.01172 (13)	0.45662 (7)	0.62604 (7)	0.0297 (3)	
N8	0.06300 (14)	0.41041 (6)	0.67314 (7)	0.0297 (3)	

### Atomic displacement parameters (Å^2^)

**Table:**

	*U*^11^	*U*^22^	*U*^33^	*U*^12^	*U*^13^	*U*^23^
C1	0.0460 (11)	0.0336 (10)	0.0415 (11)	−0.0041 (8)	0.0091 (9)	0.0020 (8)
C2	0.0366 (9)	0.0282 (9)	0.0253 (9)	−0.0023 (7)	0.0103 (7)	−0.0010 (7)
C3	0.0339 (9)	0.0289 (9)	0.0299 (9)	0.0062 (7)	0.0068 (7)	0.0019 (7)
C4	0.0271 (8)	0.0341 (10)	0.0293 (9)	0.0008 (7)	0.0053 (7)	0.0004 (7)
C5	0.0300 (8)	0.0257 (9)	0.0221 (8)	−0.0015 (7)	0.0071 (7)	−0.0002 (7)
C6	0.0296 (9)	0.0305 (9)	0.0262 (9)	0.0022 (7)	0.0046 (7)	0.0006 (7)
C7	0.0286 (8)	0.0323 (9)	0.0279 (9)	−0.0017 (7)	0.0051 (7)	−0.0011 (7)
C8	0.0342 (9)	0.0347 (10)	0.0359 (10)	0.0009 (8)	0.0009 (8)	0.0022 (8)
C9	0.0283 (8)	0.0291 (9)	0.0266 (9)	−0.0001 (7)	0.0075 (7)	0.0002 (7)
C10	0.0287 (8)	0.0275 (9)	0.0272 (9)	−0.0016 (7)	0.0081 (7)	0.0002 (7)
C11	0.0283 (8)	0.0291 (9)	0.0268 (9)	−0.0033 (7)	0.0062 (7)	−0.0013 (7)
C12	0.0321 (9)	0.0356 (10)	0.0334 (10)	−0.0063 (7)	−0.0017 (8)	0.0007 (8)
C13	0.0526 (12)	0.0315 (10)	0.0438 (11)	0.0017 (9)	0.0084 (9)	0.0024 (8)
C14	0.0298 (8)	0.0279 (9)	0.0337 (10)	−0.0026 (7)	0.0088 (7)	−0.0003 (8)
C15	0.0289 (9)	0.0337 (10)	0.0251 (9)	0.0002 (7)	0.0043 (7)	0.0031 (7)
C16	0.0291 (8)	0.0314 (9)	0.0251 (9)	−0.0016 (7)	0.0059 (7)	−0.0033 (7)
C17	0.0216 (8)	0.0270 (9)	0.0284 (9)	−0.0012 (7)	0.0063 (7)	0.0005 (7)
C18	0.0334 (9)	0.0335 (10)	0.0245 (9)	−0.0035 (7)	0.0050 (7)	0.0016 (7)
C19	0.0406 (10)	0.0310 (10)	0.0297 (9)	−0.0067 (8)	0.0088 (8)	−0.0058 (8)
C20	0.0500 (12)	0.0304 (10)	0.0517 (12)	0.0022 (8)	0.0084 (10)	0.0011 (9)
C21	0.0407 (10)	0.0255 (9)	0.0280 (9)	0.0004 (8)	0.0095 (8)	−0.0002 (7)
C22	0.0304 (9)	0.0302 (9)	0.0325 (9)	0.0057 (7)	0.0049 (7)	0.0012 (7)
C23	0.0269 (8)	0.0284 (9)	0.0326 (9)	−0.0006 (7)	0.0057 (7)	0.0009 (7)
C24	0.0287 (8)	0.0237 (8)	0.0238 (8)	0.0018 (7)	0.0072 (7)	0.0001 (7)
C25	0.0266 (8)	0.0317 (9)	0.0287 (9)	−0.0004 (7)	0.0038 (7)	−0.0023 (7)
C26	0.0327 (9)	0.0293 (9)	0.0376 (10)	−0.0053 (7)	0.0083 (8)	−0.0024 (8)
C27	0.0327 (9)	0.0315 (9)	0.0300 (9)	−0.0036 (7)	0.0025 (7)	−0.0013 (7)
C28	0.0269 (8)	0.0265 (9)	0.0278 (9)	−0.0003 (7)	0.0077 (7)	−0.0008 (7)
C29	0.0287 (8)	0.0265 (9)	0.0269 (9)	0.0006 (7)	0.0073 (7)	−0.0003 (7)
C30	0.0307 (9)	0.0277 (9)	0.0305 (9)	0.0032 (7)	0.0066 (7)	−0.0006 (7)
C31	0.0383 (10)	0.0376 (10)	0.0358 (10)	0.0066 (8)	−0.0003 (8)	0.0017 (8)
C32	0.0454 (11)	0.0294 (10)	0.0439 (11)	−0.0024 (8)	0.0027 (9)	0.0003 (8)
C33	0.0266 (8)	0.0282 (9)	0.0344 (10)	0.0009 (7)	0.0080 (7)	0.0005 (7)
C34	0.0328 (9)	0.0282 (9)	0.0291 (9)	0.0034 (7)	0.0068 (7)	0.0052 (7)
C35	0.0310 (9)	0.0318 (9)	0.0249 (9)	0.0021 (7)	0.0049 (7)	0.0012 (7)
C36	0.0231 (8)	0.0258 (9)	0.0298 (9)	0.0006 (7)	0.0067 (7)	−0.0009 (7)
C37	0.0270 (8)	0.0294 (9)	0.0282 (9)	0.0000 (7)	0.0029 (7)	0.0047 (7)
C38	0.0291 (9)	0.0344 (10)	0.0258 (9)	−0.0011 (7)	0.0025 (7)	−0.0006 (7)
N1	0.0297 (7)	0.0274 (8)	0.0306 (8)	0.0009 (6)	0.0054 (6)	−0.0002 (6)
N2	0.0315 (7)	0.0290 (8)	0.0282 (8)	−0.0009 (6)	0.0071 (6)	−0.0003 (6)
N3	0.0287 (7)	0.0325 (8)	0.0277 (8)	0.0006 (6)	0.0019 (6)	0.0021 (6)
N4	0.0240 (7)	0.0283 (8)	0.0272 (7)	0.0000 (6)	0.0015 (6)	0.0011 (6)
N5	0.0262 (7)	0.0268 (7)	0.0275 (7)	0.0024 (6)	0.0021 (6)	0.0013 (6)
N6	0.0299 (7)	0.0307 (8)	0.0297 (8)	0.0030 (6)	0.0001 (6)	−0.0009 (6)
N7	0.0305 (7)	0.0282 (8)	0.0310 (8)	−0.0003 (6)	0.0077 (6)	0.0004 (6)
N8	0.0311 (7)	0.0257 (8)	0.0324 (8)	0.0018 (6)	0.0067 (6)	0.0012 (6)

### Geometric parameters (Å, °)

**Table:**

C1—C2	1.505 (2)	C20—H20B	0.9800
C1—H1A	0.9800	C20—H20C	0.9800
C1—H1B	0.9800	C21—C22	1.381 (2)
C1—H1C	0.9800	C21—C26	1.400 (2)
C2—C7	1.392 (2)	C22—C23	1.381 (2)
C2—C3	1.398 (2)	C22—H22	0.9500
C3—C4	1.382 (2)	C23—C24	1.385 (2)
C3—H3	0.9500	C23—H23	0.9500
C4—C5	1.391 (2)	C24—C25	1.390 (2)
C4—H4	0.9500	C24—N5	1.4277 (18)
C5—C6	1.396 (2)	C25—C26	1.381 (2)
C5—N1	1.4250 (18)	C25—H25	0.9500
C6—C7	1.376 (2)	C26—H26	0.9500
C6—H6	0.9500	C27—C28	1.494 (2)
C7—H7	0.9500	C27—H27A	0.9800
C8—C9	1.494 (2)	C27—H27B	0.9800
C8—H8A	0.9800	C27—H27C	0.9800
C8—H8B	0.9800	C28—N5	1.3557 (18)
C8—H8C	0.9800	C28—C29	1.382 (2)
C9—N3	1.3241 (19)	C29—N7	1.3912 (18)
C9—C10	1.421 (2)	C29—C30	1.421 (2)
C10—C11	1.380 (2)	C30—N6	1.3226 (19)
C10—N2	1.3949 (18)	C30—C31	1.489 (2)
C11—N4	1.3513 (18)	C31—H31A	0.9800
C11—C12	1.496 (2)	C31—H31B	0.9800
C12—H12A	0.9800	C31—H31C	0.9800
C12—H12B	0.9800	C32—C33	1.505 (2)
C12—H12C	0.9800	C32—H32A	0.9800
C13—C14	1.506 (2)	C32—H32B	0.9800
C13—H13A	0.9800	C32—H32C	0.9800
C13—H13B	0.9800	C33—C34	1.392 (2)
C13—H13C	0.9800	C33—C38	1.400 (2)
C14—C15	1.382 (2)	C34—C35	1.380 (2)
C14—C19	1.398 (2)	C34—H34	0.9500
C15—C16	1.382 (2)	C35—C36	1.390 (2)
C15—H15	0.9500	C35—H35	0.9500
C16—C17	1.390 (2)	C36—C37	1.389 (2)
C16—H16	0.9500	C36—N8	1.4281 (18)
C17—C18	1.388 (2)	C37—C38	1.377 (2)
C17—N4	1.4265 (18)	C37—H37	0.9500
C18—C19	1.381 (2)	C38—H38	0.9500
C18—H18	0.9500	N1—N2	1.2620 (16)
C19—H19	0.9500	N3—N4	1.3877 (16)
C20—C21	1.505 (2)	N5—N6	1.3818 (16)
C20—H20A	0.9800	N7—N8	1.2646 (17)
			
C2—C1—H1A	109.5	C22—C21—C26	117.12 (14)
C2—C1—H1B	109.5	C22—C21—C20	121.78 (15)
H1A—C1—H1B	109.5	C26—C21—C20	121.08 (15)
C2—C1—H1C	109.5	C21—C22—C23	122.21 (15)
H1A—C1—H1C	109.5	C21—C22—H22	118.9
H1B—C1—H1C	109.5	C23—C22—H22	118.9
C7—C2—C3	118.06 (14)	C22—C23—C24	119.67 (15)
C7—C2—C1	120.87 (15)	C22—C23—H23	120.2
C3—C2—C1	121.06 (15)	C24—C23—H23	120.2
C4—C3—C2	120.96 (15)	C23—C24—C25	119.68 (14)
C4—C3—H3	119.5	C23—C24—N5	120.75 (13)
C2—C3—H3	119.5	C25—C24—N5	119.54 (13)
C3—C4—C5	120.15 (15)	C26—C25—C24	119.55 (15)
C3—C4—H4	119.9	C26—C25—H25	120.2
C5—C4—H4	119.9	C24—C25—H25	120.2
C4—C5—C6	119.40 (14)	C25—C26—C21	121.73 (15)
C4—C5—N1	116.46 (13)	C25—C26—H26	119.1
C6—C5—N1	124.04 (14)	C21—C26—H26	119.1
C7—C6—C5	119.86 (15)	C28—C27—H27A	109.5
C7—C6—H6	120.1	C28—C27—H27B	109.5
C5—C6—H6	120.1	H27A—C27—H27B	109.5
C6—C7—C2	121.55 (15)	C28—C27—H27C	109.5
C6—C7—H7	119.2	H27A—C27—H27C	109.5
C2—C7—H7	119.2	H27B—C27—H27C	109.5
C9—C8—H8A	109.5	N5—C28—C29	106.05 (14)
C9—C8—H8B	109.5	N5—C28—C27	126.45 (14)
H8A—C8—H8B	109.5	C29—C28—C27	127.48 (14)
C9—C8—H8C	109.5	C28—C29—N7	121.34 (14)
H8A—C8—H8C	109.5	C28—C29—C30	106.27 (13)
H8B—C8—H8C	109.5	N7—C29—C30	132.38 (14)
N3—C9—C10	110.37 (14)	N6—C30—C29	110.19 (14)
N3—C9—C8	119.84 (14)	N6—C30—C31	119.92 (14)
C10—C9—C8	129.75 (14)	C29—C30—C31	129.85 (15)
C11—C10—N2	121.59 (14)	C30—C31—H31A	109.5
C11—C10—C9	106.22 (13)	C30—C31—H31B	109.5
N2—C10—C9	132.15 (14)	H31A—C31—H31B	109.5
N4—C11—C10	106.25 (14)	C30—C31—H31C	109.5
N4—C11—C12	125.10 (14)	H31A—C31—H31C	109.5
C10—C11—C12	128.56 (14)	H31B—C31—H31C	109.5
C11—C12—H12A	109.5	C33—C32—H32A	109.5
C11—C12—H12B	109.5	C33—C32—H32B	109.5
H12A—C12—H12B	109.5	H32A—C32—H32B	109.5
C11—C12—H12C	109.5	C33—C32—H32C	109.5
H12A—C12—H12C	109.5	H32A—C32—H32C	109.5
H12B—C12—H12C	109.5	H32B—C32—H32C	109.5
C14—C13—H13A	109.5	C34—C33—C38	118.10 (14)
C14—C13—H13B	109.5	C34—C33—C32	121.09 (14)
H13A—C13—H13B	109.5	C38—C33—C32	120.81 (15)
C14—C13—H13C	109.5	C35—C34—C33	120.89 (15)
H13A—C13—H13C	109.5	C35—C34—H34	119.6
H13B—C13—H13C	109.5	C33—C34—H34	119.6
C15—C14—C19	117.42 (15)	C34—C35—C36	120.50 (15)
C15—C14—C13	121.53 (15)	C34—C35—H35	119.8
C19—C14—C13	121.04 (15)	C36—C35—H35	119.8
C14—C15—C16	122.26 (15)	C37—C36—C35	119.15 (14)
C14—C15—H15	118.9	C37—C36—N8	124.70 (14)
C16—C15—H15	118.9	C35—C36—N8	116.12 (14)
C15—C16—C17	119.32 (14)	C38—C37—C36	120.27 (15)
C15—C16—H16	120.3	C38—C37—H37	119.9
C17—C16—H16	120.3	C36—C37—H37	119.9
C18—C17—C16	119.68 (14)	C37—C38—C33	121.10 (15)
C18—C17—N4	119.24 (14)	C37—C38—H38	119.5
C16—C17—N4	121.08 (14)	C33—C38—H38	119.5
C19—C18—C17	119.90 (15)	N2—N1—C5	113.85 (13)
C19—C18—H18	120.0	N1—N2—C10	115.00 (13)
C17—C18—H18	120.0	C9—N3—N4	105.28 (12)
C18—C19—C14	121.37 (15)	C11—N4—N3	111.87 (12)
C18—C19—H19	119.3	C11—N4—C17	129.93 (13)
C14—C19—H19	119.3	N3—N4—C17	118.04 (12)
C21—C20—H20A	109.5	C28—N5—N6	111.74 (12)
C21—C20—H20B	109.5	C28—N5—C24	130.61 (13)
H20A—C20—H20B	109.5	N6—N5—C24	117.65 (12)
C21—C20—H20C	109.5	C30—N6—N5	105.72 (12)
H20A—C20—H20C	109.5	N8—N7—C29	114.72 (13)
H20B—C20—H20C	109.5	N7—N8—C36	113.94 (13)
			
C7—C2—C3—C4	−0.2 (2)	N7—C29—C30—C31	2.1 (3)
C1—C2—C3—C4	178.96 (14)	C38—C33—C34—C35	−0.2 (2)
C2—C3—C4—C5	0.5 (2)	C32—C33—C34—C35	178.94 (14)
C3—C4—C5—C6	−1.1 (2)	C33—C34—C35—C36	0.3 (2)
C3—C4—C5—N1	−177.62 (13)	C34—C35—C36—C37	0.1 (2)
C4—C5—C6—C7	1.3 (2)	C34—C35—C36—N8	−177.74 (13)
N1—C5—C6—C7	177.54 (13)	C35—C36—C37—C38	−0.6 (2)
C5—C6—C7—C2	−0.9 (2)	N8—C36—C37—C38	177.05 (14)
C3—C2—C7—C6	0.4 (2)	C36—C37—C38—C33	0.7 (2)
C1—C2—C7—C6	−178.76 (14)	C34—C33—C38—C37	−0.3 (2)
N3—C9—C10—C11	0.47 (17)	C32—C33—C38—C37	−179.43 (14)
C8—C9—C10—C11	−177.43 (15)	C4—C5—N1—N2	−165.00 (13)
N3—C9—C10—N2	178.31 (15)	C6—C5—N1—N2	18.6 (2)
C8—C9—C10—N2	0.4 (3)	C5—N1—N2—C10	−177.65 (12)
N2—C10—C11—N4	−178.96 (13)	C11—C10—N2—N1	−176.78 (14)
C9—C10—C11—N4	−0.84 (17)	C9—C10—N2—N1	5.6 (2)
N2—C10—C11—C12	−2.3 (3)	C10—C9—N3—N4	0.10 (16)
C9—C10—C11—C12	175.78 (15)	C8—C9—N3—N4	178.24 (13)
C19—C14—C15—C16	−0.1 (2)	C10—C11—N4—N3	0.96 (17)
C13—C14—C15—C16	−179.32 (14)	C12—C11—N4—N3	−175.82 (14)
C14—C15—C16—C17	−1.7 (2)	C10—C11—N4—C17	176.29 (13)
C15—C16—C17—C18	2.1 (2)	C12—C11—N4—C17	−0.5 (3)
C15—C16—C17—N4	−178.76 (13)	C9—N3—N4—C11	−0.66 (16)
C16—C17—C18—C19	−0.8 (2)	C9—N3—N4—C17	−176.61 (12)
N4—C17—C18—C19	−179.95 (13)	C18—C17—N4—C11	−141.55 (16)
C17—C18—C19—C14	−1.0 (2)	C16—C17—N4—C11	39.3 (2)
C15—C14—C19—C18	1.4 (2)	C18—C17—N4—N3	33.53 (19)
C13—C14—C19—C18	−179.32 (15)	C16—C17—N4—N3	−145.59 (14)
C26—C21—C22—C23	0.5 (2)	C29—C28—N5—N6	1.63 (17)
C20—C21—C22—C23	−177.80 (15)	C27—C28—N5—N6	−176.99 (14)
C21—C22—C23—C24	−2.1 (2)	C29—C28—N5—C24	−178.40 (13)
C22—C23—C24—C25	2.0 (2)	C27—C28—N5—C24	3.0 (3)
C22—C23—C24—N5	179.86 (13)	C23—C24—N5—C28	29.9 (2)
C23—C24—C25—C26	−0.3 (2)	C25—C24—N5—C28	−152.21 (15)
N5—C24—C25—C26	−178.23 (13)	C23—C24—N5—N6	−150.15 (14)
C24—C25—C26—C21	−1.3 (2)	C25—C24—N5—N6	27.76 (19)
C22—C21—C26—C25	1.2 (2)	C29—C30—N6—N5	0.50 (16)
C20—C21—C26—C25	179.52 (15)	C31—C30—N6—N5	178.53 (13)
N5—C28—C29—N7	179.31 (12)	C28—N5—N6—C30	−1.35 (16)
C27—C28—C29—N7	−2.1 (2)	C24—N5—N6—C30	178.68 (12)
N5—C28—C29—C30	−1.24 (17)	C28—C29—N7—N8	−167.46 (14)
C27—C28—C29—C30	177.37 (15)	C30—C29—N7—N8	13.3 (2)
C28—C29—C30—N6	0.46 (18)	C29—N7—N8—C36	−178.99 (12)
N7—C29—C30—N6	179.83 (15)	C37—C36—N8—N7	9.3 (2)
C28—C29—C30—C31	−177.31 (16)	C35—C36—N8—N7	−172.99 (13)

### Hydrogen-bond geometry (Å, °)

**Table:**

Cg1 and Cg2 are the centroids of the C33–C38 benzene ring and the N4/N3/C9–C11 pyrazole ring, respectively.

**Table:**

*D*—H···*A*	*D*—H	H···*A*	*D*···*A*	*D*—H···*A*
C7—H7···Cg1^i^	0.95	2.76	3.4847 (18)	133
C27—H27A···Cg2^ii^	0.98	2.72	3.6322 (18)	156

Symmetry codes: (i) −*x*+1, −*y*+1, −*z*+1; (ii) −*x*, −*y*+1, −*z*+1.
